# Innovative Stem Cell Assisted Lipotransfer Approach for Breast Reconstruction in cancer Patients: Efficacy and Safety of the LIPO MULTI-SVF Study

**DOI:** 10.1007/s12015-025-10921-9

**Published:** 2025-07-02

**Authors:** Francesco Agostini, Miriam Marangon, Stefania Zanolin, Marco Valvasori, Naike Casagrande, Martina Urbani, Elisabetta Lombardi, Valentina Visintini Cividin, Cristina Durante, Mario Mazzucato, Samuele Massarut

**Affiliations:** 1 Stem Cell Unit, Centro di Riferimento Oncologico di Aviano (CRO) IRCCS, Via F. Gallini, 2, Aviano, 33081 PN Italy; 2 Unit of Molecular Oncology and Preclinical Model of Cancer Progression, Centro di Riferimento Oncologico di Aviano (CRO) IRCCS, Aviano, Italy; 3 Cancer Radiology Unit, Centro di Riferimento Oncologico di Aviano (CRO) IRCCS, Aviano, Italy; 4 Breast Cancer Unit, Centro di Riferimento Oncologico di Aviano (CRO) IRCCS, Aviano, Italy

**Keywords:** Breast cancer, Reconstructive Medicine, Cell-assisted Lipotransfer, Stromal Vascular Fraction, Validated/standardized Isolation Procedure, Staggered Administration, Ultrasound Imaging, Safety, Efficacy, Longitudinal single-arm Clinical Study

## Abstract

**Supplementary Information:**

The online version contains supplementary material available at 10.1007/s12015-025-10921-9.

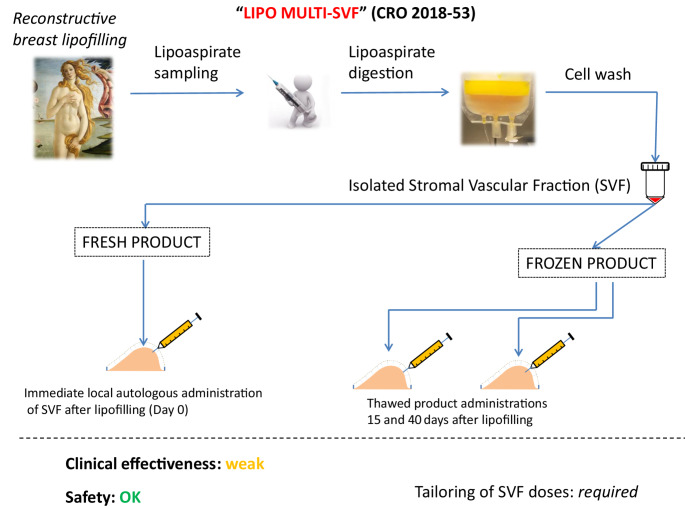

Dear Editor,

cell-assisted lipotransfer (CAL) is a breast reconstruction technique that enhances graft persistence compared to standard lipofilling interventions [1]. In such approach, the adipose filler is enriched with stromal vascular fraction (SVF) cells, isolated from the adipose tissue [2], which support tissue homeostasis. However, a previous study in mice showed that excessive SVF enrichment impaired fat graft retention [2]. Furthermore, SVF cells are almost completely lost within 4 weeks post-transplantation [3]. Therefore, optimizing a safe and effective CAL method is necessary. To address these challenges, we tested the hypothesis that distributing SVF cells over time might improve graft persistence and prevent cell competition and necrosis. The LIPO MULTI-SVF clinical trial (phase I/II) was conducted in accordance with the Declaration of Helsinki (2004). The study was approved by the Ethics Committee of the CRO Aviano National Cancer Institute (protocol code: CRO-2018-53; approval date: November 11, 2018). All patients signed an informed consent upon enrollment. Enrolled female patients (Fig. 1a) (*n* = 12; age 52.6 ± 1.0 years; body mass index 23.7 ± 0.7) underwent breast reconstruction via standard lipofilling, following mastectomy due to breast cancer. Half of the harvested lipoaspirate was used for SVF isolation in an authorized clean room, as previously described [4]. A portion of the cell product (approximately one-third) was administered (fresh) to the patient immediately after the procedure, while the remaining cells were frozen [4]. Thawed aliquots were administered after 15 ± 2 days (A15) or 40 ± 2 days (A40) post-intervention. Patients met the following criteria: low recurrence risk (age 49–59 years; cancer subtype: non-triple negative and non-HER2 positive; stage: <3), and a body mass index sufficient to harvest > 200 ml of lipoaspirate. Clinical exclusion criteria included positivity for anti-HIV-Ab, anti-HCV-Ab, HBsAg, syphilis test, and beta-HCG. Safety was evaluated by assessing pain, inflammation/infection, and the occurrence of liponecrosis or sebaceous cysts via ultrasound imaging. Clinical effectiveness was evaluated by measuring adipose graft thickness at 9 detection points using ultrasound. Assessments were performed at baseline (*n* = 12), at short/medium-term (15 ± 2 days and 40 ± 2 days; *n* = 12), and during follow-up at 6 months (*n* = 9, safety; *n* = 3, effectiveness) and 1 year (*n* = 8, safety; *n* = 2, effectiveness) post-lipofilling.

The adipose filler volume was 160.8 ± 17 ml, while the starting material for SVF isolation was 134.8 ± 16.4 ml. The mean cell count was 393.5 ± 23.4 nucleated cells/ml of lipoaspirate, with a cell viability of 78.7 ± 2%. Flow cytometry analysis revealed that 38.9 ± 3.7% of the SVF nucleated cells were CD45^−^/CD34^+^/CD31^−^. The primary CFU-F frequency exceeded 1% in all fresh SVF samples, while the secondary CFU-F frequency was 25.4 ± 0.06%. Adequate differentiation potential into adipocytes and osteocytes was demonstrated for all tested samples (Fig. 1b). The total number of cells in the aliquots used for immediate (A0) or delayed (A15 and A40) SVF administrations was 10.7 ± 1.1 × 10⁶ and 14.3 ± 1.6 × 10⁶, respectively. After one week of freezing, the viability of nucleated cells was 71.9 ± 1.7%. Sterility tests were positive for *Staphylococcus epidermidis* in 2 cases out of 36, both sampled after the administration of A15 and A40 aliquots (*n* = 24). The mean thickness of the transplanted adipose graft (Fig. 1c) was significantly greater 15 days post-lipofilling compared to baseline (One-way ANOVA *p* = 0.025; Tukey’s HSD *post hoc p < 0.05*). No significant reduction in thickness was observed 40 days post-lipofilling versus 15 days. However, adipose graft thickness progressively decreased at 6 months and 1 year after lipofilling. Supplementary Figure S1 provides thickness measurements obtained at baseline, 15 and 40 days post-lipofilling, and after 6 months and 1 year of follow-up. No acute toxicity due to SVF administration was observed. One patient experienced vasovagal syncope after the A40 administration, but this event was considered unrelated to the procedure. No short-term clinical signs of local inflammation, infection, or liponecrosis were observed, as confirmed by ultrasound imaging. During the 6-month follow-up, one patient developed axillary adenopathies, though no pathological findings were evidenced. Six patients underwent breast implant or tissue expander replacement due to inflammation or capsular contracture; none of these events were linked to SVF administration. After 1 year and at the current time (i.e., 2.0–5.8 years of median follow-up), no breast cancer relapses or secondary malignancies have been reported.

Restoring body image after mastectomy is essential for psychosocial recovery and may also aid in cancer treatment. This study assessed the safety and potential clinical effectiveness of a novel CAL method, in which three staggered aliquots of SVF cells (A0, A15, and A40) were administered into the grafted adipose tissue. The results showed that the efficiency of cell collection, viability of obtained SVF cells, their phenotype, and differentiation potential were consistent with international guidelines [2]. In the short term, after the administration of SVF aliquots, no signs of adipose filler resorption were observed, which aligns with other studies demonstrating the short-term benefits of CAL in breast reconstruction [5]. However, our approach did not show a long-term benefit. Excessive cell crowding within the transplanted graft can be ruled out due to the study design; therefore, it is likely that the amount of SVF administered was insufficient to sustain long-term graft homeostasis. Given the promising results observed at short term, future research should focus on refining the timing and dosage of SVF administration to enhance long-term graft retention and improve clinical outcomes for breast reconstruction patients. This reconstructive procedure did not result in adverse events or worsened pain or inflammation parameters. As no infections were observed in any patient, the positive sterility tests reported after product administration were likely related to sampling technique. After a 1-year follow-up, and as of the submission of this manuscript (i.e., 2.0–5.8 years), no neoplastic recurrences have been reported. The staggered distribution of SVF cells over time in the fat graft appears to be a key feature improving the safety of our approach.

In conclusion, while the study demonstrated short-term safety and feasibility, the importance of long-term monitoring is essential to fully assess the potential risks and benefits of this novel approach. Thus, the present study paves the way for the optimization of CAL techniques, providing a novel approach that could potentially improve the long-term success of breast reconstruction.

**Fig. 1:**
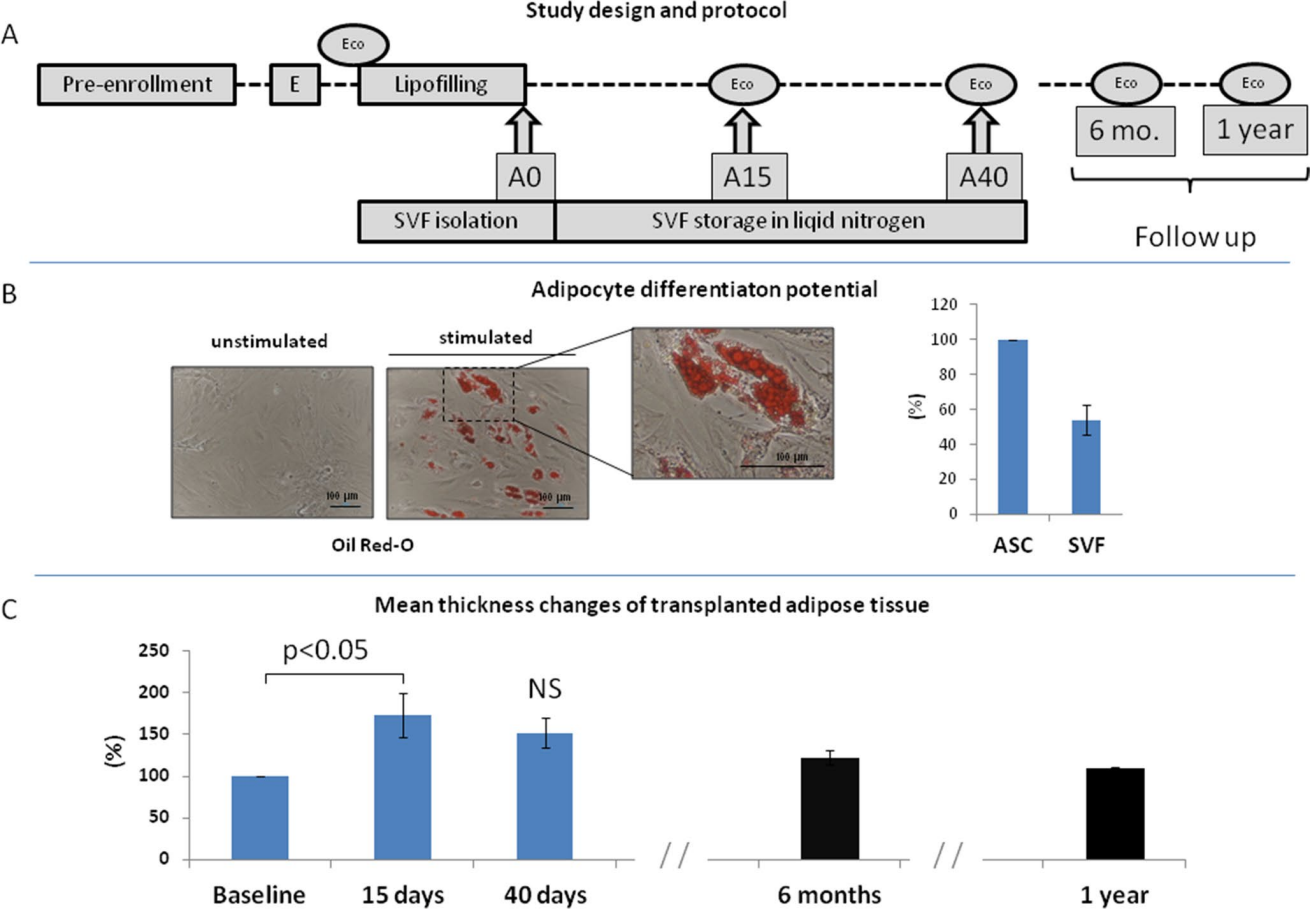
**a** Illustrates a schematic representation of the entire study design. The clinical inclusion and exclusion criteria were assessed during the "Pre-enrollment" phase (Fig. 1). Upon enrollment (E), patients signed informed consent forms. Prior to lipofilling, patients underwent ultrasonographic evaluations (Eco) to assess baseline tissue thickness at specific anatomical sites in the breast. During the standard lipofilling procedure, stromal vascular fraction (SVF) was isolated from half of the collected lipoaspirate. Fresh SVF, representing approximately one-third of the total cell product, was administered immediately following the lipofilling intervention (A0). The remaining portion of the cell product was frozen until further administration. Thawed SVF aliquots were then administered to patients, at the doctor's office, 15±2 days (A15) and 40±2 days (A40) after the initial lipofilling. Additional Eco evaluations were performed immediately before the A15 and A40 administrations of SVF. Follow-up Eco measurements were conducted at 6 and 12 months (Mo) post-lipofilling. **b** Shows the differentiation potential of SVF cells after transient culture. The adipogenic differentiation potential of SVF cells was compared to an equal number of commercially available adipose mesenchymal stem/stromal cells (ASC), in which adipocyte differentiation was induced using the same technique. The adipocyte differentiation potential of ASC was considered the reference value (100%). **c** Depicts the mean percentage change in adipose graft thickness. Pre-lipofilling measurements (Baseline) were compared (as percentage change) to the mean thickness of the breast-transplanted adipose tissue, as assessed by ultrasonographic imaging at different time points (refer to the Experimental Design section). These included measurements taken 15±2 and 40±2 days post-intervention, as well as 6 months and 1 year after the procedure. One-way ANOVA for repeated measures was used to assess the significance of differences between the mean measurements taken at Baseline, 15 days, and 40 days post-lipofilling (p=0.025). Tukey’s HSD was used for post hoc analysis. The results indicated that the mean thickness measured at 15±2 days was significantly greater than the baseline values. However, no significant differences (NS) were observed in the mean thickness changes between the measurements taken at 40±2 days and those at 15±2 days. Follow-up measurements taken at 6 and 12 months, from a smaller subset of patients, did not show significant differences from baseline values

## Electronic Supplementary Material

Below is the link to the electronic supplementary material.

Supplementary Material 1

## Data Availability

No datasets were generated or analysed during the current study.
