# Supplementary material for: Innovative Stem Cell Assisted Lipotransfer Approach for Breast Reconstruction in cancer Patients: Efficacy and Safety of the LIPO MULTI-SVF Study
Source: Stem Cell Rev Rep. 2025 Jul 2;21(7):2339–42. doi: 10.1007/s12015-025-10921-9 (PMC12408722; doi:10.1007/s12015-025-10921-9)

Article title: "Innovative stem cell assisted lipotransfer approach for breast reconstruction in cancer patients: efficacy and safety of the LIPO MULTI-SVF study."

Journal name: Stem Cell Reviews and Reports

Author names: Francesco Agostini[1*], Miriam Marangon[1], Stefania Zanolin[1], Marco Valvasori[1], Naike Casagrande[2], Martina Urbani[3], Elisabetta Lombardi[1], Valentina Visintini Cividin[4], Cristina Durante[1], Mario Mazzucato[1] and Samuele Massarut[4]

Affiliation:
[1]Stem Cell Unit, Centro di Riferimento Oncologico di Aviano (CRO) IRCCS, Aviano, Italy
[2]Unit of Molecular Oncology and Preclinical Model of Cancer Progression, Centro di Riferimento Oncologico di Aviano (CRO) IRCCS, Aviano, Italy
[3]Cancer Radiology Unit, Centro di Riferimento Oncologico di Aviano (CRO) IRCCS, Aviano, Italy
[4]Breast Cancer Unit, Centro di Riferimento Oncologico di Aviano (CRO) IRCCS, Aviano, Italy

e-mail address of the corresponding author: fagostini@cro.it

Supplementary Figure S1

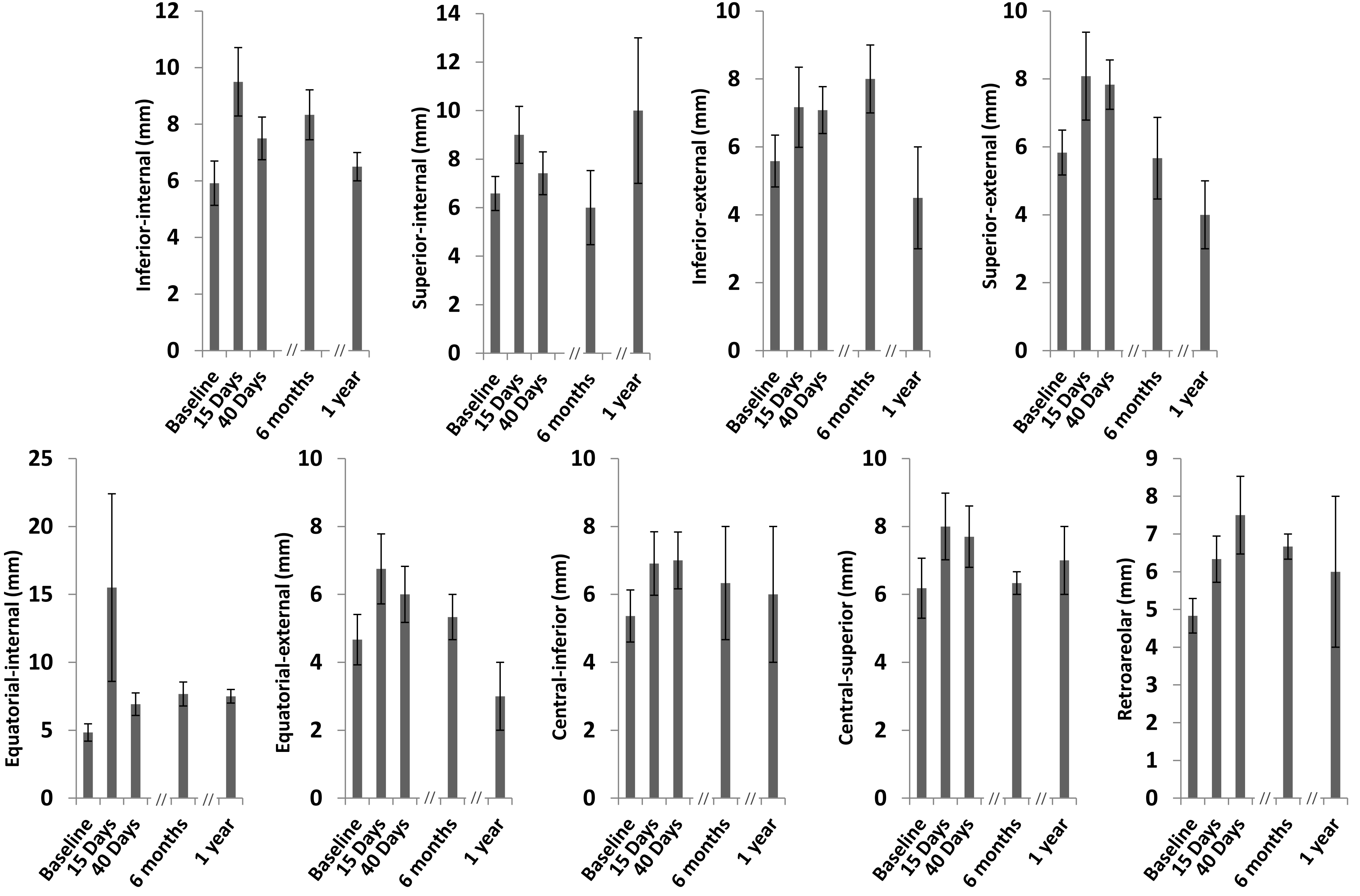

Supplement: Supplementary file 1 — Supplementary Material 1 [file 12015_2025_10921_MOESM1_ESM.pdf]
